# Application Value of the Motor Unit Number Index in Patients With Kennedy Disease

**DOI:** 10.3389/fneur.2021.705816

**Published:** 2021-12-21

**Authors:** Shuo Zhang, Xin Yang, Yingsheng Xu, Yongmei Luo, Dongsheng Fan, Xiaoxuan Liu

**Affiliations:** ^1^Department of Neurology, Peking University Third Hospital, Beijing, China; ^2^Department of Neurology, Changchun Central Hospital, Changchun, China; ^3^Beijing Municipal Key Laboratory of Biomarker and Translational Research in Neurodegenerative Diseases, Beijing, China

**Keywords:** motor unit number index, motor unit size index, Kennedy's disease, compound muscle action potential, spinal and bulbar muscular atrophy functional rating scale, MUNIX sum score

## Abstract

The aim of this study was to evaluate the usefulness of the motor unit number index (MUNIX) technique in Kennedy disease (KD) and test the correlation between the MUNIX and other clinical parameters. The MUNIX values of the bilateral deltoid, abductor digiti minimi (ADM), quadriceps femoris (QF), and tibialis anterior (TA) were determined and compared with the course of the disease. The MUNIX sum score was calculated by adding the MUNIX values of these 8 muscles. Disability was evaluated using the spinal and bulbar muscular atrophy functional rating scale (SBMAFRS). The MUNIX scores of patients with KD were negatively correlated with the course of the disease (*p* < 0.05), whereas their motor unit size index (MUSIX) scores were positively correlated with the course the of disease (*p* < 0.05). MUNIX sum scores were correlated with SBMAFRS scores (*r* = 0.714, *p* < 0.05). MUNIX was more sensitive than compound muscle action potentials or muscle strength as an indicator of neuron loss and axonal collateral reinnervation. The MUNIX sum score is an objective and a reliable indicator of disease progression, and it is a potential choice for therapeutic clinical trials. The MUNIX can assess the functional loss of motor axons and is correlated with disability. The MUNIX sum score may be especially suitable as an objective parameter.

## Introduction

Kennedy's disease (KD), also known as X-linked recessive spinal and bulbar muscular atrophy (X-SBMA), is a rare, late-onset, slowly progressive X-linked recessive neuromuscular disease with an incidence of ~1/400,000 (1). The clinical manifestation is characterized by weakness and atrophy localized proximally in the limbs, bulbar involvement, and abnormalities of the sensory nerves and endocrine system (2). The basic pathological feature of KD is the degeneration of anterior horn motor neurons and dorsal root ganglion cells (3). KD may have similar symptoms to amyotrophic lateral sclerosis (ALS), including morbidity due to weakness, but KD has a greater zchronic progression rate with a fairly normal lifespan. A good biomarker is needed to reflect both neuron loss and collateral reinnervation.

Electromyographic studies play an important role in the diagnosis of KD but are not appropriate for monitoring disease progression or for use as outcome measures in therapeutic trials (4). Earlier studies have shown that disability in KD is linked to axonal loss (5). Neither muscle strength nor the amplitude of compound muscle action potentials (CMAPs) directly reflects the change in motor neuron loss in KD, probably due to chronic compensatory collateral sprouting (6, 7).

In recent years, motor unit number estimation (MUNE) has been increasingly used in research on the pathogenesis, diagnosis, progression, drug response, and prognosis of neuromuscular diseases, particularly ALS (8, 9). The traditional MUNE techniques include the incremental technique (incr-MUNE), the statistical method (stat-MUNE), multiple point stimulation (mps-MUNE) (10, 11), spike-triggered averaging (STA) MUNE, high-density surface electromyography (EMG) decomposition MUNE, Bayesian MUNE, and MScanFit (12–14). However, the traditional MUNE method has the disadvantages of being time-consuming, unsuitable for proximal muscles and not highly repeatable. A computer-based method for estimating the motor unit number index (MUNIX) provides a new, rapid, non-invasive method for counting motor units and evaluating residual motor neurons (15). The MUNIX is widely applied in multifocal motor neuropathy (16), chronic inflammatory demyelinating polyneuropathy (CIDP) (17), and ALS (18–20). In addition to the mentioned neuromuscular diseases, MUNIX has also been applied to different categories, such as stroke, spinal cord injury, and cerebral palsy (21–24). To our knowledge, the MUNIX technique has not been studied in KD.

The purpose of this work was to investigate the change in MUNIX and explore whether the MUNIX sum score could be a sensitive biomarker to reflect the progression of KD. The MUNIX technique was applied in patients with KD and healthy control subjects to provide insight into the underlying mechanism of the change in denervation and reinnervation of lower motor neurons in KD.

## Methods

### Subjects

Thirty patients with KD and 30 healthy controls were enrolled in this study from May 2017 to August 2020 at Peking University Third Hospital. Patients with KD were genetically diagnosed with CAG repeat sequences > 40 in the first exon of the androgen receptor (AR) gene (25). All patients with KD were men, aged 47–74 (53.6 ± 5.3) years. The course of disease was between 3 and 15 years, with an average course of disease of 5.26 years. All healthy subjects were men aged 47–70 (51.6 ± 6.4) years. There was no significant difference in age between patients with KD and control subjects (*p* > 0.05). Peripheral neuropathy, diabetes, cervical spondylosis and trauma were excluded in all subjects (peripheral nerve tests, including sensory and motor nerve conduction of the bilateral median, ulnar, common peroneal, tibial, and sural nerves, were normal in all control subjects). All subjects signed an informed consent form and were approved by the Ethics Committee of Peking University Third Hospital (2019-003-02).

### Clinical Examinations

All patients with KD were examined by at least 2 experienced neurologists. Family history, clinical features, muscle strength, and the SBMA functional rating scale (SBMAFRS) (26) were recorded in great detail. Muscle strength was measured through manual testing using the Medical Research Council (MRC) scale in deltoid, abductor digiti minimi (ADM), quadriceps femoris (QF), and tibialis anterior (TA). The SBMAFRS was specifically designed for patients with KD in 2015. It consists of 14 items, each of which contains 5 (0–4) alternatives. The 14 items were divided into 5 subscales: bulbar, upper limb, trunk, lower limb, and breathing. Possible total scores range from 0 (worst) to 56 (normal) (26).

### MUNIX Analysis and Nerve Conduction Velocity Studies

A Danish Keypoint G4 instrument made by Natus was used with a MUNIX-specific operating interface. The parameters were as follows: scanning speed 3 ms/D; sensitivity 2 mV/D; filter 5 Hz−5 kHz; recording length 200 ms; internal trigger; repeat frequency 3 Hz; disposable self-sealing Ag/AgCl surface electrode; supine position; and room temperature 25°C.

The MUNIX and the motor unit size index (MUSIX) values of the bilateral deltoid, ADM, QF, and TA were measures. The MUNIX sum score was calculated by adding the results of bilateral deltoid, ADM, QF, and TA. The MUNIX studies were performed by the same technician with the same device.

**Figure 1:**
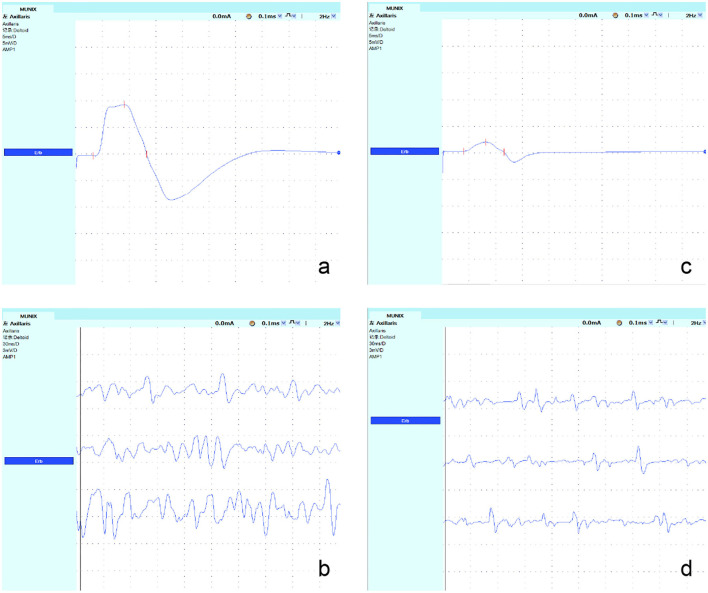
Recordings from the axillary nerves in a representative control subject and a patient with KD. **(a,c)** The maximum CMAP amplitude was obtained at Erb's point, and the negative peak amplitude was measured. The CMAP amplitude of the axillary nerve decreased significantly in patients with KD. **(b,d)** The SIP was obtained; its value was decreased significantly in patients with KD.

**Figure 2:**
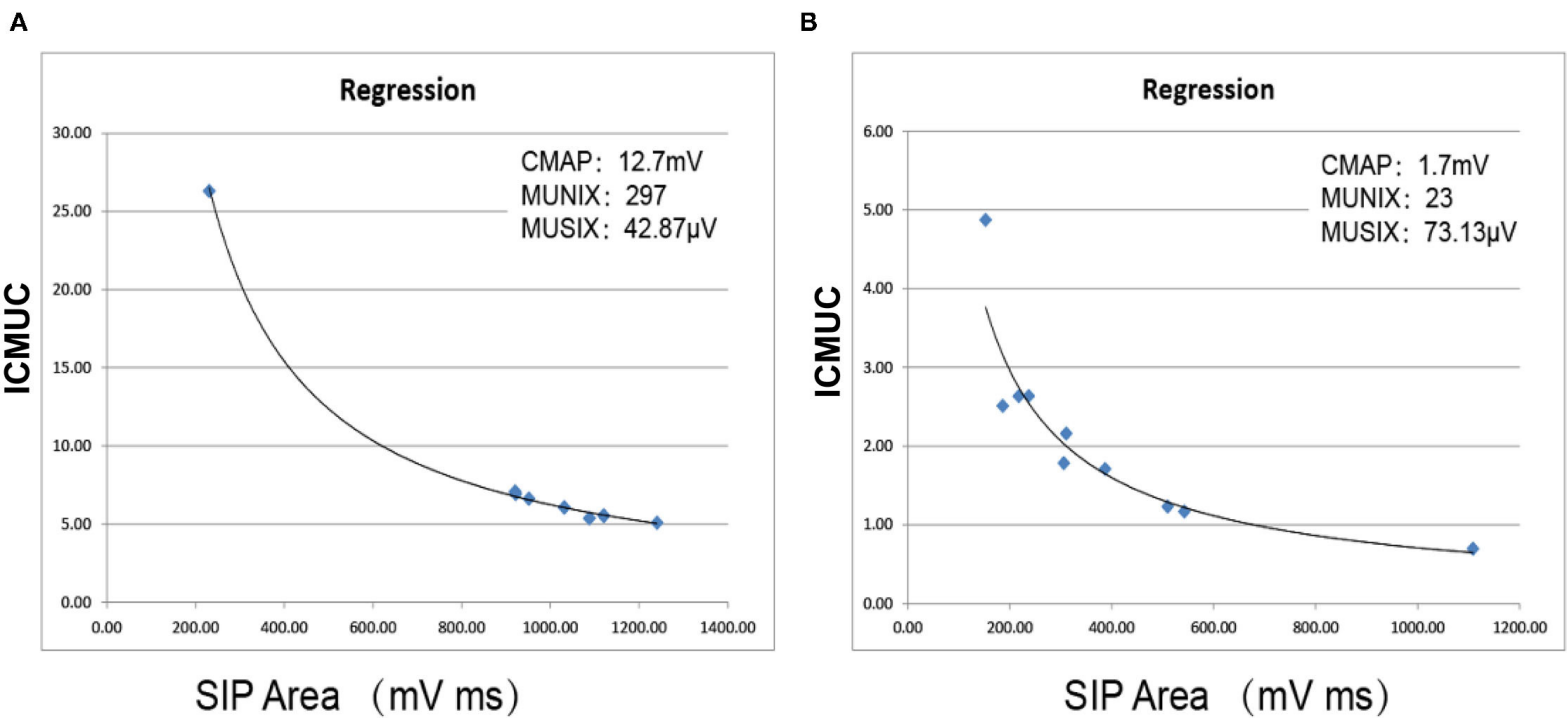
Automatic calculation of MUNIX and MUSIX values using mathematical functions. **(A)** Right QF in the control subject. **(B)** Right QF in a KD patient. ICMUC, ideal case motor unit count; SIP, surface EMG interference pattern.

The MUNIX technique has three steps for specific testing (27):

1. Step 1: The surface electrode was attached to each muscle to complete the maximum CMAP and the negative peak (minimum rise time and sharp negative takeoff) amplitude. The axillary nerves, ulnar nerves, femoral nerves, and peroneal nerves were measured. The recording electrodes were placed over the ADM, TA, and deltoid muscles using the standard belly-tendon method. During the recordings of QF, active electrodes were placed in the vastus medialis 30 cm under the stimulating site of the femoral nerve at the line of the medial border of the patella, and reference electrodes were placed distal to 3 cm. The stimulation points were Erb's point for the deltoid muscle, the ulnar nerve at the wrist for the ADM, the femoral nerve at the level of inguinal ligament for the QF, and the peroneal nerve at the fibular head for the TA. The skin temperature was kept between 34 and 36°C.
2. Step 2: The surface electromyography interference pattern (SIP) was obtained. The subjects were told to actively contract their muscles. A total of 10 levels of data were collected (ten isometric contractions that ranged from ~10 to 100% of maximal strength were assessed by the counteracting force given by the technician and by the amplitude and the fullness of the SIP). The voluntary tasks for the muscles were arm abduction for deltoid, abduction of the fifth finger for the ADM, knee extension for the QF, and foot dorsiflexion for the TA. The protocol was repeated three times for each subject. Finally, the whole range of SIPs with stable signals was measured (Figure 1).
3. Step 3: The negative peak amplitude of CMAP and the SIP scores were processed in an Excel table (Microsoft Office). The MUNIX and MUSIX scores were automatically calculated by the device through mathematical functions [an SIP epoch must fulfill three criteria: 1. SIP area > 20 mV/ms; 2. ideal case motor unit count (ICMUC) < 100; and 3. SIP area/CMAP area > 1] (27) (Figure 2).

### Statistical Analysis

Statistical analysis was performed using the SPSS 19.0 software package. Quantitative data are expressed as the mean ± standard deviation (*x* ± s). If these subjects did not meet the normal distribution, the non-parametric Mann–Whitney U test was used. The enumeration data were expressed as ratios, and the chi-square test was used. Pearson correlation coefficients were used for correlation analysis. A two-sided *p*-value < 0.05 was considered significant. By calculating the mean and standard deviation, a 95% confidence interval (CI) was obtained. In this work, the results of the second order polynomial fitting are used, which is more common. Its equations and coefficients are shown in Figures 4, 5.

## Results

### Comparison of MUNIX, MUSIX, and CMAP in the KD Group and Healthy Controls

**Figure 3:**
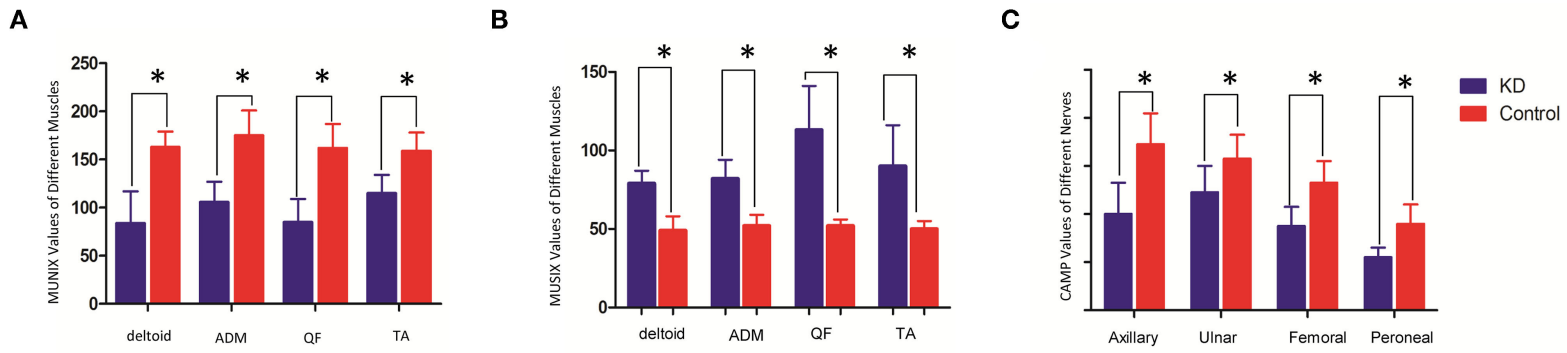
Comparison of MUNIX values **(A)**, MUSIX values **(B)** (unit: μV) and CMAP amplitudes **(C)** (unit: mV) between the KD group and the healthy control group (x ± s). *Represents a statistically significant difference.

The mean MUNIX scores of deltoid, ADM, QF, and TA in the KD group were 84 ± 33, 106 ± 21, 85 ± 24, and 115 ± 19, respectively. They were all significantly lower than those of control subjects (*p* < 0.05). The mean MUSIX scores of the affected muscles were 79 ± 8, 82 ± 12,113 ± 28, and 90 ± 26 μV. They are all significantly higher than the control group (*p* < 0.05). The negative peak amplitude of CMAP of the corresponding nerve are all significantly lower than the control group (*p* < 0.05) (Table 1; Figure 3). The SNAP amplitudes of the ulnar nerves and sural nerves, as well as the sensory nerve conduction velocities, were also significantly lower than those of the control group (*p* < 0.05).

**Table 1:** Comparison of MUNIX, MUSIX, and CMAP values in patients with KD and control subjects.

**Muscles**	**Parameter**	**KD patients (*n* = 30)**	**Control subjects** **(*n* = 30)**	***p*-value**
Deltoid	MUNIX	84 ± 33	163 ± 16	*p =* 0.038
	MUSIX (μV)	79 ± 8	49 ± 9	*p =* 0.033
	CMAP (mV)	4.0 ± 1.3	6.9 ± 1.3	*p =* 0.042
	Muscle strength (MRC scale)	3.5 ± 0.9	—	—
ADM	MUNIX	106 ± 21	175 ± 26	*p =* 0.045
	MUSIX (μV)	82 ± 12	52 ± 7	*p =* 0.035
	CMAP (mV)	4.9 ± 1.1	6.3 ± 1.0	*p =* 0.050
	Muscle strength (MRC scale)	4.1 ± 0.7	—	—
QF	MUNIX	85 ± 24	162 ± 25	*p =* 0.044
	MUSIX (μV)	113 ± 28	52 ± 4	*p =* 0.034
	CMAP (mV)	3.5 ± 0.8	5.3 ± 0.9	*p =* 0.040
	Muscle strength (MRC scale)	3.6 ± 1.0	—	—
TA	MUNIX	115 ± 19	159 ± 19	*p =* 0.043
	MUSIX (μV)	90 ± 26	50 ± 5	*p =* 0.043
	CMAP (mV)	2.2 ± 0.4	3.6 ± 0.8	*p =* 0.046
	Muscle strength (MRC scale)	4.1 ± 0.6	—	—
Deltoid+ ADM+	MUNIX	781.9 ± 159.8	235.0 ± 66.4	*p* < 0.001
QF +TA sum score	MUSIX (μV)	728.3 ± 124.3	405.4 ± 17.2	*p* < 0.01
	CMAP (mV)	27.5 ± 6.7	44.2 ± 2.9	*p* = 0.023
	MRC scale	30.5 ± 5.0	—	—

*KD, kennedy disease; MUNIX, motor unit number index; MUSIX, motor unit size index; CMAP, compound muscle action potential; ADM, abductor digiti minimi; QF, quadriceps femoris; TA, tibialis anterior*.

The abnormal sensory nerve action potential (SNAP) amplitude of 17 ulnar nerves in patients with KD was also compared with the MUNIX and MUSIX scores of ADM. There was no significant difference between the SNAP decrease and MUNIX (*p* > 0.05).

The values of MUNIX, MUSIX, and CMAP of four left and right muscles were compared in patients with KD. There was no significant difference (*p* > 0.05) (Table 2) between the two groups.

**Table 2:** Comparison of the MUNIX, MUSIX, and CMAP values of four bilateral muscles in patients with KD (x ± s).

**Muscles**	**Parameter**	**Left (*n* = 30)**	**Right (*n* = 30)**	***p*-value**
Deltoid	MUNIX	81 ± 30	83 ± 26	*p =* 0.058
	MUSIX (μV)	74 ± 6	69 ± 9	*p =* 0.102
	CMAP (mV)	3.8 ± 1.1	3.9 ± 1.2	*p =* 0.081
ADM	MUNIX	103 ± 20	105 ± 21	*p =* 0.137
	MUSIX (μV)	80 ± 10	82 ± 9	*p =* 0.125
	CMAP (mV)	4.5 ± 1.2	4.3 ± 1.0	*p =* 0.088
QF	MUNIX	84 ± 21	82 ± 23	*p =* 0.156
	MUSIX (μV)	110 ± 25	112 ± 24	*p =* 0.089
	CMAP (mV)	3.3 ± 0.7	3.3 ± 0.9	*p =* 0.074
TA	MUNIX	111 ± 17	113 ± 16	*p =* 0.092
	MUSIX (μV)	88 ± 23	85 ± 25	*p =* 0.077
	CMAP (mV)	2.1 ± 0.5	2.2 ± 0.6	*p =* 0.103

*Data are shown in the mean ± standard deviation. A two-sided p-value > 0.05 was not considered significant*.

*KD, kennedy disease; MUNIX, motor unit number index; MUSIX, motor unit size index; CMAP, compound muscle action potential; ADM, abductor digiti minimi; QF, quadriceps femoris; TA, tibialis anterior*.

### Abnormality Rate

Abnormality of a parameter is defined as a value greater than the mean plus 1.95 x SD. The normal limit values of the deltoid, ADM, QF, and TA, respectively, were as follows: MUNIX <131.8, <124.3, <113.3, <122.0; MUSIX >66.6, >65.7, >59.8, >59.8 μV; CMAP <4.4, <4.3, <3.5, <2.0. Abnormal rates of MUNIX scores in the KD groups: 47 (78%) deltoid, 45 (75%) ADM, 48 (80%) QF, and 40 (67%) TA. Abnormality rates of MUSIX scores: 49 (82%) deltoid, 46 (76%) ADM, 54 (90%) QF, and 41 (68%) TA. Abnormality rates of CMAP amplitude: 29 (48%) axillary nerves, 22 (37%) ulnar nerves, 31 (52%) femoral nerves, and 21 (35%) peroneal nerves. Abnormality rates of SNAP amplitude: 17 (28%) ulnar nerves and 22 (37%) sural nerves. Abnormality rate of sensory conduction velocity: 18 (30%) ulnar nerves and 23 (38%) sural nerves. A significantly higher frequency of abnormalities in MUNIX and MUSIX than in CMAP was observed in patients with KD (deltoid: *X*^2^ = 20.08, *p* < 0.05, ADM: *X*^2^ = 19.33, *p* < 0.05, QF: *X*^2^ = 23.21, *p* < 0.05, TA: *X*^2^ = 23.10, *p* < 0.05). Of the tested muscles, QF was the most severely affected, which was consistent with the clinical observations.

### The Correlation Between MUNIX and Disease Duration and Muscle Strength

The MUNIX scores of each muscle correlated with strength in the deltoid, ADM, QF, and TA (*r* = 0.585, *p* < 0.01; *r* = 0.735, *p* < 0.01; *r* = 0.701, *p* < 0.01; *r* = 0.712, *p* < 0.01). MUNIX scores also correlated significantly with the CMAP of the corresponding nerves (*r* = 0.681, *p* = 0.045; *r* = 0.648, *p* = 0.048; *r* = 0.699, *p* = 0.042; *r* = 0.687, *p* = 0.042). The MUNIX sum score correlated significantly with the SBMAFRS score (35.7 ± 5.1) (*r* = 0.714, *p* < 0.01). The MUSIX sum score correlated significantly with the SBMAFRS score (35.7 ± 5.1) (*r* = −0.782, *p* < 0.05).

**Figure 4:**
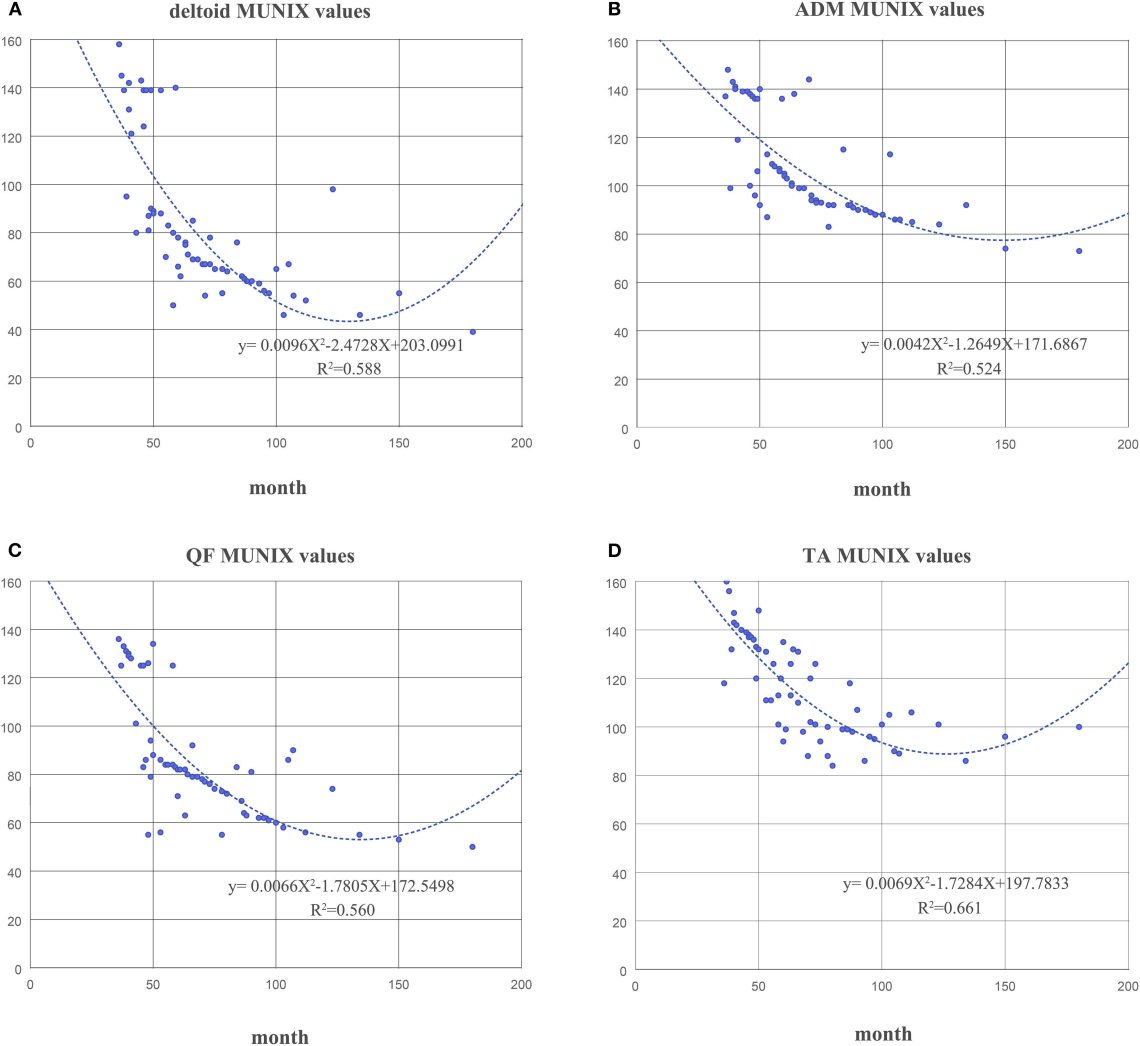
The correlation between the course of disease and the MUNIX scores of the deltoid **(A)**, ADM **(B)**, QF **(C)**, and TA **(D)** in the KD group.

**Figure 5:**
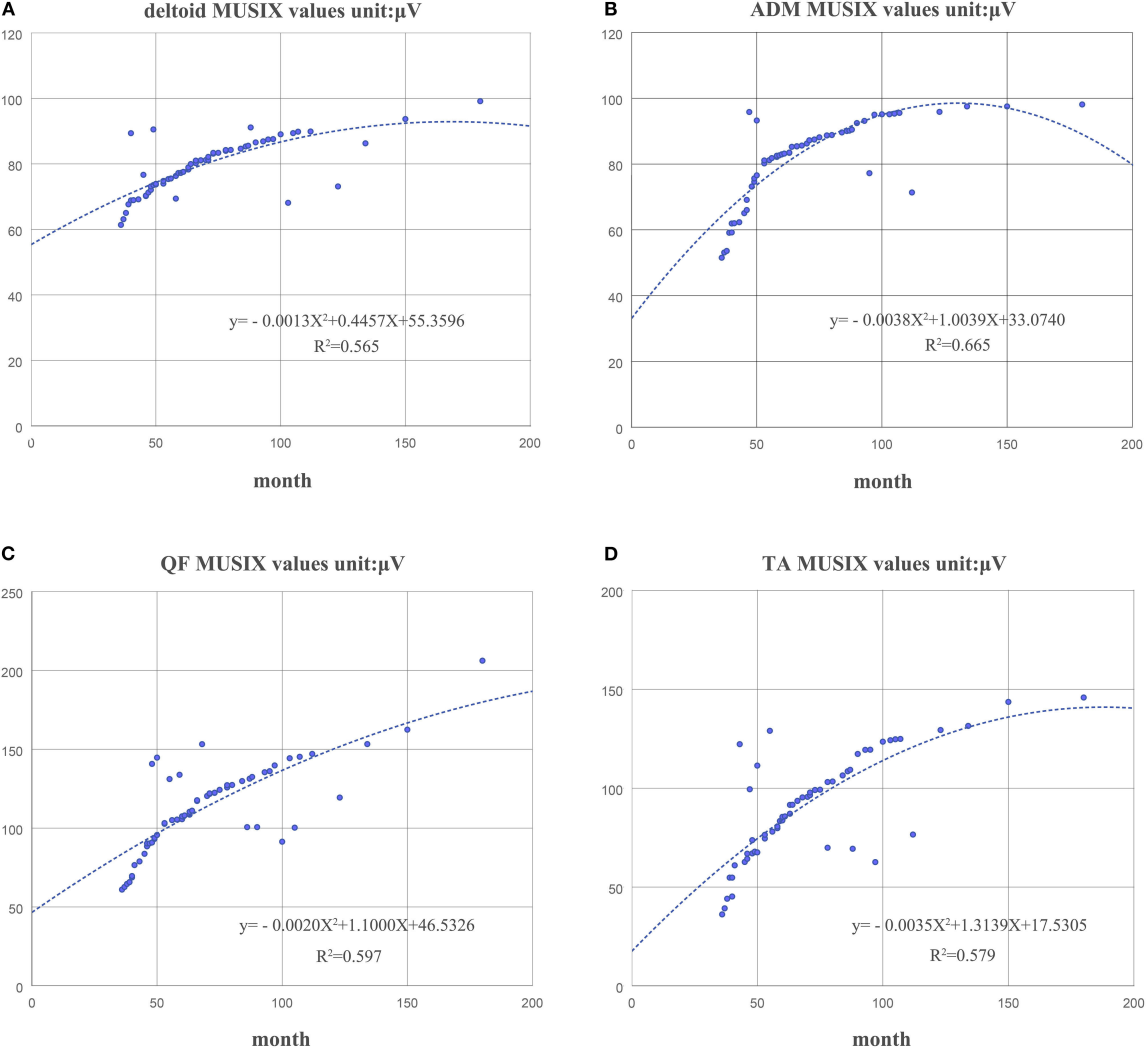
The correlation between the course of disease and the MUSIX scores of the deltoid **(A)**, ADM **(B)**, QF **(C)**, and TA **(D)** in the KD group.

The duration of disease in the KD group was negatively correlated with the MUNIX scores of each muscle (deltoid, *r* = −0.826, *p* < 0.01; ADM, *r* = −0.777, *p* < 0.01; QF, *r* = −0.746, *p* < 0.01 and TA, *r* = −0.799, *p* < 0.01) (Figure 4). However, it was positively correlated with the MUSIX scores of the deltoid, ADM, QF, and TA (*r* = 0.756, *p* < 0.01; *r* = 0.834, *p* < 0.01; *r* = 0.744, *p* < 0.01; *r* = 0.737, *p* < 0.01) (Figure 5).

## Discussion

In this study, we explored the feasibility and usefulness of the MUNIX technique in KD. As expected, our findings demonstrated that the MUNIX values of the deltoid, ADM, QF, and TA muscles were significantly lower in patients with KD than in control subjects due to motor neuron loss. The MUSIX value of the 8 muscles was significantly higher since collateral sprouted from the remaining axon in these muscles. These results were in agreement with those found in ALS patients (9), indicating that MUNIX and MUSIX could be reliable indicators of changes in motor neuron loss and chronic motor axonal innervation and reinnervation. This is consistent with clinical observations. Of all the tested muscles, QF was the most severely affected. To our knowledge, there are no current studies of MUNIX in KD.

We chose to perform MUNIX assessment on the bilateral, the deltoid, ADM, QF, and TA because these muscles could reflect changes in the proximal and distal muscles. The most prominent change was seen in QF muscles. This reflects the observed clinical patterns of early wasting of the proximal muscle in the lower limb. A significant decrease in MUNIX and increase in MUSIX in the QF muscle were found compared with other affected muscles. A recent clinical trial used the MUNIX of the ADM and TA as additional biomarker endpoints (28). Our results demonstrated that the MUNIX values of proximal muscle may be more appropriate to disable changes in the disease. In contrast to ALS, most patients with KD present relatively bilateral symmetrical weakness and atrophy (29). There was no significant difference between different lateral muscles in MUNIX and MUSIX (30). Therefore, we can draw a bold hypothesis that four muscles can be used instead of eight muscles to reflect the relationship between the course of disease and functional score in future research.

The reason why we did not choose the foot muscles was that their CMAP amplitude was usually lower in patients with KD. We observed that the errors were significantly increased in individual patients with severe muscle atrophy, especially when the CMAP amplitude was <0.5 mV. These errors have been reported in other studies (31). Therefore, the MUNIX evaluation requires an accurate CMAP amplitude above 0.5 mV. It was reported that proximal muscles were not easy to assess, as the obtained stimulation was often uncomfortable compared with other muscles (9). However, in our study, patients with KD had good compliance and high reproducibility in the proximal nerves. Although we did not show the reproducibility of MUNIX values, many previous studies have shown good reproducibility with MUNIX compared with other traditional MUNE techniques (9, 18).

In our work, the abnormality rates of MUNIX values were significantly higher than those of CMAP amplitudes. The MUNIX technique can reflect the clinical change in lower motor neurons earlier than the routine electrophysiological technique (32, 33). Similar results were also shown in patients with ALS (34). The decrease in MUNE value was more prominent than the reduction in CMAP amplitude, which may be partly due to collateral sprouting in the CMAP amplitude of the corresponding nerve (4). Therefore, MUNE values were more sensitive than CMAP amplitude in monitoring neuron loss (35). In this work, MUNIX values and CMAP amplitude had good correlations. Both parameters were significantly correlated with the strength of those muscles. MUNIX protocols include the MUSIX score, which can evaluate the size of motor units and further reflect the degree of collateral reinnervation. One limitation of MUNE is that it cannot record axonal reinnervation in ALS (32). MUNIX protocols can overcome pitfalls and provide more information than CMAP alone, not only regarding motor neuron loss but also regarding axonal reinnervation, which is especially suitable for slowly progressive disease in which the CMAP amplitude of the corresponding nerve is relatively preserved in KD.

Our work showed that MUNIX values correlated with muscle strength and CMAP amplitude. Since MUNIX scores closely depend on CMAP amplitude, which of the muscles is especially influenced by the placement of the electrodes, an experienced technician is essential (36). The sum of the results of deltoid, ADM, QF, and TA resulted in the MRC sum score and the MUNIX sum score. Our study did not compare SBMAFRS with MUNIX of each muscle because SBMAFRS is a whole-body evaluation, and we merely compared the sum scores. A work showed that the MUNIX sum score correlated with the SBMAFRS with a lower *p*-value. The MUNIX sum score included electrophysiological evaluation of the proximal and distal muscle of limbs with more objective and more reliable results. There was a more significant difference than MUNIX of each muscle alone during the comparison between controls and patients. MUNIX scores have also been used in other neuromuscular diseases. In ALS, motor disability and the ALS functional rating scale score have been evaluated by MUNIX (34). In CIDP, the MUNIX sum score correlated with clinical scores. Thus, the MUNIX sum score is an objective and reliable indicator to reflect the progression of the disease, suggesting that it may be a potential choice for therapeutic clinical trials.

Our findings showed that there was no significant difference between the amplitude of abnormal SNAP and MUNIX scores of corresponding muscles in patients with KD. This may be related to AR staining active inclusion bodies, the dense aggregation of aggregates in the nucleus of motor neurons, or the aggregation of sensory neurons scattered in the cytoplasm (37). It may also result from different mechanisms of damage to sensory and motor neurons.

Quantification of motor unit number has long been of clinical and scientific significance as it relates to monitoring disease progression and/or assessing the effects of pharmacologic and behavioral interventions on motor unit numbers (38). This work shows that the MUNIX score and sum score can assess motor neuron loss, as well as collateral reinnervation. As suggested in ALS, it may be a good technique to evaluate the progression rate of patients with KD. It is expected that MUNIX can be added in future follow-up studies and may be considered a complementary test for KD after routine EMG detection.

Some studies indicate that there are potential limitations in the application of MUNIX methods in atrophied muscle, where it is unclear whether atrophy is accompanied by loss of motor units or loss of muscle fiber size. Nonetheless, the findings from the sensitivity analysis provided by this work still offer valuable guidance in predicting the trend of changes in the MUNIX estimates, with variation in different motor unit properties (39). The results of the myopathy study indicates that MUNE values obtained in myopathic patients could not necessarily accurately reflect the population of functioning motor units. MUNIX may potentially present this same kind of pitfall, as indeed the researcher observed a significant decline in its values in clinically affected muscles from myopathic patients. In their work, MUSIX did not change significantly and could be used to help determine whether the MUNIX decrease is indeed accompanied by motor neuron loss. Thus, although it is a marker of disease progression with proven value in primarily denervating disorders, it is recommendable to consider the underlying clinical context beforehand, as it could potentially influence the analysis/interpretation of MUNIX results (40). There are also studies on stroke patients, which indicates that it remains a dilemma to apply the MUNIX technique in stroke patients. They advocate the application of a range of techniques (together with standard MUNIX (sMUNIX) or modified MUNIX (mMUNIX) measurement) to the same patients with stroke (rather than solely relying on one technique) to obtain more definite information. These techniques (such as quantitative analysis of motor unit action potentials, muscle fiber density analysis, and electrical impedance myography) can address different aspects of the examined muscle and thus offer a significant amount of complementary information about muscle structure and function. This is important for further improvement of the MUNIX method as well as for appropriate application and interpretation of MUNIX measures in different diseases or situations (41).

## Data Availability Statement

The original contributions presented in the study are included in the article/supplementary material, further inquiries can be directed to the corresponding author/s.

## Ethics Statement

This study was approved by the Ethics Committee of Peking University Third Hospital (2019-003-02). The patients/participants provided their written informed consent to participate in this study.

## Author Contributions

XL, DF, and SZ: study design. SZ and XY: experimental implementation and data collection. SZ, XY, YX, and YL: data analysis and manuscript drafting. All authors designed the experiment and approved the final version of the paper.

## Funding

This study was supported by the National Natural Science Foundation of China (81873784).

## Conflict of Interest

The authors declare that the research was conducted in the absence of any commercial or financial relationships that could be construed as a potential conflict of interest.

## Publisher's Note

All claims expressed in this article are solely those of the authors and do not necessarily represent those of their affiliated organizations, or those of the publisher, the editors and the reviewers. Any product that may be evaluated in this article, or claim that may be made by its manufacturer, is not guaranteed or endorsed by the publisher.
